# Insights into the binding of Ag ions with SilE model peptides: an NMR and MS coupled approach

**DOI:** 10.1093/mtomcs/mfad015

**Published:** 2023-03-13

**Authors:** Gabriele Antonio Zingale, Valentina Oliveri, Giuseppe Grasso

**Affiliations:** IRCCS-Fondazione Bietti, Rome, Italy; Department of Chemical Sciences, University of Catania, Catania, Italy; Department of Chemical Sciences, University of Catania, Catania, Italy

**Keywords:** SilE, Nuclear Magnetic Resonance (NMR), Mass Spectrometry (MS), Chemical Shift Perturbation (CSP), Peptides, Proteins, Silver Resistance, Metal complex

## Abstract

The diffuse and renewed use of silver as antimicrobial agent has caused the development of resistance to silver ions in some bacterial strains, posing a serious threat for health systems. In order to cast light on the mechanistic features of resistance, here, we aimed to understand how silver interacts with the periplasmic metal-binding protein SilE which is engaged in bacterial silver detoxification. This aim was addressed by studying two peptide portions of SilE sequence (SP2 and SP3) that contain the putative motifs involved in Ag^+^ binding. We demonstrate that SP2 model peptide is involved in silver binding through its histidine and methionine residues in the two HXXM binding sites. In particular, the first binding site is supposed to bind the Ag^+^ ion in a linear fashion, while the second binding site complexes the silver ion in a distorted trigonal planar fashion. We propose a model where the SP2 peptide binds two silver ions when the concentration ratio Ag^+^/SP2 is ≥10.0. We also suggest that the two binding sites of SP2 have different affinity for silver. This evidence comes from the change in the path direction of the Nuclear Magnetic Resonance (NMR) cross-peaks upon the addition of Ag^+^. Here, we report the conformational changes of SilE model peptides occurring upon silver binding, monitored at a deep level of molecular details. This was addressed by a multifaceted approach, combining NMR, circular dichroism, and mass spectrometry experiments.

## Graphical Abstract

**Graphical Abstract:**
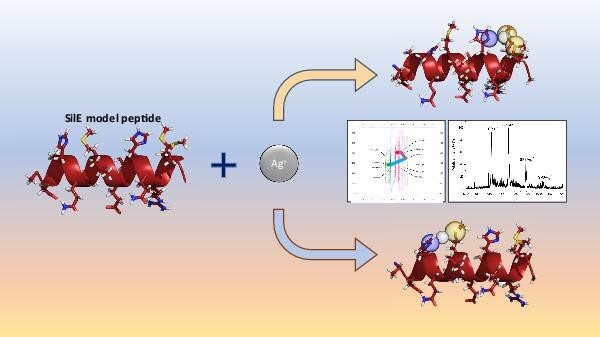
Model peptides of the silver-binding periplasmic protein SilE and their interaction with Ag^+^ investigated by Nuclear Magnetic Resonance and Mass Spectrometry.

## Introduction

Some microorganisms, like bacteria and protozoa, have gained resistance toward antimicrobial agents. This leads to the inefficacy of the treatment with such agents, allowing the microorganism to spread and infect multiple sites and organs, further increasing the risk of transmission and diffusion across the population. In 1975, the extensive use of silver sulfadiazine as an antimicrobial agent in various formulations for different clinical applications spanning from surgery, dentistry, and ophthalmology (i.e. contact lens preservation) led to the discovery of silver-resistant bacteria from burn units at the Massachusetts General Hospital where three of the first cases died of septicemia after infection by multiply resistant *Salmonella typhimurium*.^1^

Membrane ATPases proteins or membrane potential-dependent cation/proton antiporter proteins constitute the main route to the development of mechanism of resistance toward toxic heavy metal because they “pump out,” by active export, the toxic ion outside of the cell.^2,3^ The plasmid pMG101 is responsible for the resistance to Ag^+^ ions in bacteria and it is approximately 180 kb in size. It is made up of nine genes divided into three transcriptional units^4^: *silE, silRS*, and *silCFBAGP*, each with its individual promoter.^5^ The product encoded by the *silE* gene is a metal-binding protein of 143 amino acids located in the periplasm and it is specific for Ag^+^.^4^ The genes *silCBA* and *silP* encode for two parallel metal ion efflux pumps, a three-component cation/proton antiporter and a membrane ATPase respectively, which are part of the resistance mechanism.^4,5^

One of the main hypotheses on how silver ions are eliminated from the bacteria is that they are seized by SilE in the periplasm and then expelled through the SilCBA complex. The silver resistance apparatus is the first example of a system with three distinct resistance components, which are encoded in only one gene cluster that seems to be transcriptionally regulated by the product of *silS* and *silR* genes.^5^

In particular, the SilE protein is present only when the *silE* gene is intact, and transcription is induced only in the presence of silver ions. Even though proteins commonly use the sulfur in cysteines as a ligand to coordinate and bind metals, SilE possesses neither cysteines nor aromatic amino acids. Nevertheless, in its sequence, SilE includes 11 methionine and 10 histidine residues, and these are the main candidates for metal binding.^4^

In previous studies, computational and experimental methods have been used to evaluate the 20 amino acids affinity for silver for a 1:1 stoichiometry. From these studies, the three basic amino acids histidine, lysine, and arginine with their side chains, are considered the strongest binders for silver ions. Instead, among the nonpolar amino acids, methionine showed the strongest affinity for Ag^+^.^6–8^ Essentially, Ag^+^ coordination in peptides can occur with the oxygen atoms of the C=O, the amide in the backbone, or with the heteroatoms in the side chain of the amino acids, depending on the peptide sequence. Asiani *et al.* defined SilE as an intrinsically disordered protein that changes its structure into a predominant α-helix after Ag^+^ selective binding. Hence, SilE protein has essentially no secondary structure and it changes its folding to an α-helical shape after Ag^+^ binding, as measured by circular dichroism (CD) and 2D NMR. CD studies showed that six Ag^+^ ions represent the optimal amount of silver ions for the full folding of SilE, but from mass spectrometry (MS) measurements it was seen that two more Ag^+^ ions can be bound by SilE for a total stoichiometry of eight silver ions per protein.^9^ The tertiary structure and the geometries of silver complexes in SilE are still unknown. However, NMR titrations of SilE fragments with Ag^+^ showed a fast chemical exchange forming a 1:1 complex. Moreover, it is very likely that methionine residues in SilE motifs participate in silver binding, this assumption confirmed by failed missed oxidation of these residues in the presence of Ag^+^.^10^

**Fig. 1:**
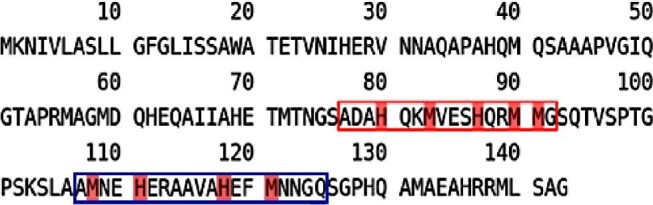
Primary structure of SilE protein. SP2 and SP3 peptides are boxed in red and blue, respectively. A highlight in red identifies the putative amino acid residues involved in Ag^+^ binding.

Therefore, as only very few studies have investigated the interaction between SilE and silver ions, in this work, we report that two model peptides of the periplasmic protein SilE can interact with silver ions through their methionine and histidine residues. The amino acid sequence of SilE is shown in Fig. 1, with a highlight on the model peptides considered in this study (SP2 and SP3). SP2 corresponds to the fragment starting from residue 77 and ending on residue 92 on the entire protein, while SP3 corresponds to the fragment starting from residue 107 and ending on residue 125. The behavior of SP2, which contains the HXXMM motif, is more complicated and the involvement of the second methionine has never been proved. Thus, we studied the interaction between SP2 and silver using CD and 2D-NMR technique to identify the Ag^+^ binding site of this model peptide. The interaction between SP3 model peptide and silver ions was investigated with CD and electrospray ionization MS (ESI–MS) to determine the stoichiometry and the binding constant of the Ag^+^/SP3 complexes. Results show that the two model peptides studied, SP2 and SP3, have two putative Ag^+^ binding sites each, and they have different coordination modes and affinities for this metal ion.

## Materials and methods

### Solid-phase peptide synthesis of SP2 and SP3

Peptides were manually synthesized using Fmoc solid-phase peptide synthesis (SPPS) on a 2-chloro-trityl 2-chloride resin chosen as the solid support (1.22 mmol/g resin loading). Cycles of amino acid coupling, washing, and deprotection are carried out until the sequence of the desired peptides is obtained. The peptides are then cleaved from the resin with a mixture of trifluoroacetic acid (TFA), triisopropylsilane, thioanisole, and water. Diethyl ether was added to precipitate the peptide and the solution was centrifuged. The pellet was redissolved in 0.1% TFA in water and 5% of acetonitrile (ACN) for high-performance liquid chromatography (HPLC) purification.

### Circular dichroism titrations of SP2 and SP3 with Ag^+^

CD experiments were conducted at the Centre of Biomolecular Sciences at Nottingham University, Nottingham, UK, with an Applied Photophysics Chirascan-Plus fitted with a Quantum Northwest temperature controller (JASCO, UK). The amino acid sequence of the SP2 and SP3 peptides are ADAHQKMVESHQRMMG (MW = 1856.115) and AMNEHERAAVAHEFMNNGQ (MW = 2156.322), respectively. For the SP2 peptide, the data were acquired at 25°C into a 1 mm path length quartz cuvette (Hellma Analytics). Solution A contained 100 μM SP2 peptide and 5 mM 4-(2-hydroxyethyl)-1-piperazineethanesulfonic acid (HEPES)/MilliQ buffer solution; the solution B, contained 100 μM SP2 peptide, 5 mM HEPES/MilliQ buffer solution, and 1 mM of silver nitrate solution. The silver nitrate to peptide concentration ratio in the second solution was 10:1. Volumes of solution A and solution B were mixed to obtain different Ag^+^/SP2 ratio for each CD experiment. Spectra were recorded from 190 to 250 nm; scan speed: 20 nm/min; response time: 1 s, with each spectrum representing an average of five accumulations, 15 scans per point. A scan of buffer alone was subtracted from the sample curves. As for SP3, CD measurements were performed in a 2 mm path length quartz cuvette on a JASCO (J-1500) CD spectropolarimeter at 25°C in the wavelength range from 190 to 250 nm at a scan speed of 20 nm/min with each spectrum representing an average of four accumulations. All the data points were baseline corrected. An aqueous solution (1.5 mM) of SP3 was prepared and properly diluted to have a 25 μM solution of the peptide in the cuvette. The peptide was titrated with a solution of AgNO_3_ (6.7 mM).

### NMR titration of SP2 with Ag^+^

Two solutions, A and B, were prepared. Solution A contained 1 mM SP2 and 5 mM HEPES. Solution B contained 1 mM SP2, 5 mM HEPES, and 10 mM AgNO_3_. The pHs of solutions A and B were adjusted to pH 7.5 with 10% NH_3_. All the 2D-NMR spectra were acquired using a Bruker 800 MHz Avance III spectrometer with QCI cryoprobe. The titration was carried out adding volumes of solution B to volumes of solution A, keeping the concentration of SP2 and HEPES constant in solution. Volumes of solution A and solution B were mixed to obtain a different Ag^+^/SP2 ratio for each NMR experiment, with a total sample volume of 600 μL.

### Chemical shift perturbation

CcpNMR Analysis V2 software was used to analyse the NMR spectra and assign the residues in the SP2 peptide. The peak labelling was performed manually and in a semi-automated way (with the help of the assignment propagation command). CCpNMR V2 was also used to follow the chemical shift movement across the titration spectra. An NMR series was created, and the chemical shifts of each cross-peak were extrapolated to calculate chemical shift perturbations (CSPs). The assignment started with the total correlation spectroscopy (TOCSY) spectrum of the pure peptide in solution. The peaks on this spectrum were high quality and facilitated a full assignment of the spectra. The peak movement on the TOCSY spectra was calculated as a CSP using the following equation. Each peak in the spectrum has an *x*-value and a *y*-value. The distance was calculated using Pythagorean theorem: $CS{P}_{ij} = \sqrt {( \Delta {\delta }_{H_ij_{(F1)}} )^2 + ( \Delta {\delta }_{H_ij_{(F2)}} )^2} $, where ∆δ_H_*_ij_* is the difference in chemical shift between the starting position on the F1 (typically on *y*-axis) or F2 (typically on *x*-axis) and the end position on F1 or F2, respectively. To measure the CSP on ^1^H-^13^C HSQC spectra, the equation was corrected with a scaling factor (α) on the ^13^C frequency axis: $CS{P}_{ij} = \sqrt {( \Delta {\delta }_{H_{ij}} )^2 + \alpha {( \Delta {\delta }_{C_{ij}} )}^2} $. The α coefficient was set to 0.3 as suggested in literature.^11^ If not specified, the starting point is the 0.0:1.0 (Ag^+^: peptide concentration) spectrum and the end point is the 10.0:1.0.

### ESI–MS titration of SP3 with Ag^+^

A 0.5 mM stock solution of SP3 was prepared by dissolving 2 mg of peptide in 2 mL of MilliQ water. A 20.0 mM stock solution of ammonium acetate (CH_3_COONH_4_) was prepared by dissolving 16.8 mg in 10.9 mL of MilliQ water. A 1.0 mM stock solution of AgNO_3_ was prepared by dilution of 44 μL AgNO_3_ 45.6 mM in 1.96 mL of MilliQ water. All the ESI–MS samples injected in the instrument were prepared from the stock solution cited above and had a total volume of 400 μL, SP3 100 μM, CH_3_COONH_4_ 9.0 mM, and pH = 7.0. They were injected into the ion source at a flow rate of 50 μL/min using nitrogen as drying gas. All ESI–MS measurements were carried out by using a Finnigan LCQ DECA XP PLUS ion-trap spectrometer operating in positive-ion mode and equipped with an orthogonal ESI source (Thermo Electron Corporation, USA).

## Result and discussion

Intrinsically disordered proteins can be studied with CD and NMR, which are excellent techniques for this purpose. CD is an extremely useful technique to study the folding, secondary structures, and/or binding properties of peptides and proteins in a rapid and efficient manner. The chromophores are the peptide bonds in the backbone of the protein that absorb in the far UV region (240–180 nm). Each CD spectrum is characteristic of a particular secondary structure.

**Fig. 2:**
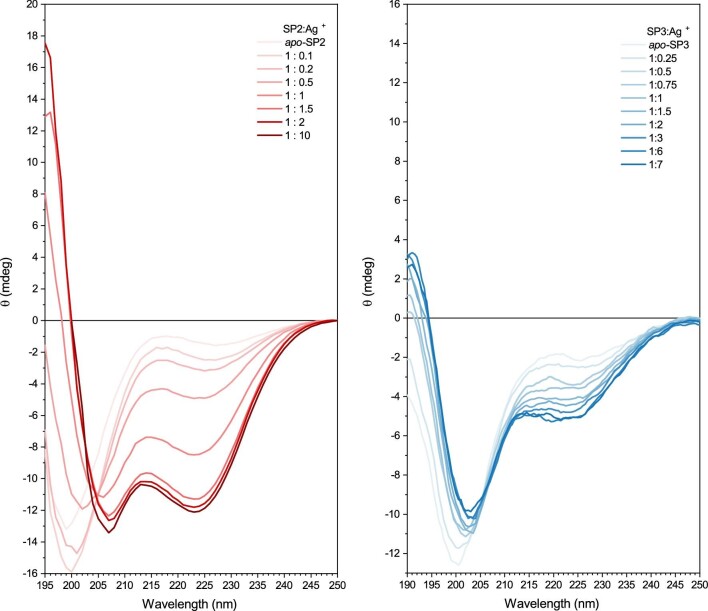
CD spectra of the SP2 (red) and SP3 (blue) peptides acquired in presence of increasing amounts of Ag^+^.

For instance, an α-helical secondary structure gives negative peaks at 222 and 208 nm and a positive peak at 193 nm.^12^ Moreover, proteins with a random coil structure have a negative peak near 195 nm.^13^ CD titration spectra collected on SP2 and SP3 upon silver binding show a change in the secondary structure of SP2 and SP3. The CD spectra of *apo*-SP2 and *apo*-SP3 in Fig. 2 have a strong negative peak around 200 nm, which is indicative of a random coil peptide arrangement. However, in both cases, the addition of Ag^+^ causes a significant change in the spectrum. As the concentration of silver in the solution increases, two negative bands at approximately 205 and 225 nm start to appear in the CD spectra. Hence, the two model peptides acquire an α-helix secondary structure in the presence of Ag^+^. However, the CD spectrum of SP2 suggests its folding into α-helix upon Ag^+^ addition, contrary to the SP3 peptide which displays a poor α-helix evolution during Ag^+^ addition and appear thus less structured in the presence of Ag^+^ (Fig. 2).

SP2 contains two Ag^+^ binding motifs, HXXM and HXXMM and the involvement of Met91 in the second one is not well defined from previous studies.^14^ Thus, the NMR experiments here conducted on SP2 were set up to assess if the Met91 is involved in the same coordination geometry as His87 and Met90, using a method based on a thorough analysis of 2D CSPs of all the signals on the 2D-NMR TOCSY and HSQC spectra.

To follow the interaction of the SP2 model peptide with Ag^+^ ions, a solution containing the biomolecule was titrated with an increasing amount of Ag^+^, as explained in the methods section. Two-dimensional NMR spectra were acquired from each titration point and the titration was monitored from 2D TOCSY and ^1^H-^13^C HSQC spectra. The high resolution of the TOCSY and ^1^H-^13^C HSQC cross-peaks has been used to undoubtedly assign all the ^1^H nuclei in the peptide. To rationalize the shift of the cross-peaks in TOCSY and ^1^H-^13^C HSQC experiments during the titration, we used the CSP technique.^11^ The analysis of CSPs is quite straightforward; peaks showing the greatest extent of movement are probably involved in the interaction between the two compounds of interest. Thus, CSPs can guide in finding the interaction site of biomolecules, especially when each peak is assigned.

CSPs in TOCSY and ^1^H-^13^C HSQC spectra were compared after an initial classification in four different groups; the criteria used are described in detail in the supplementary information. Mean and standard deviation (σ) were calculated for each group and a further discrimination in weak, medium, and strong movers was then based on the CSP value (supplementary information). Because similar peptides change their secondary structure from random coil to α-helix upon Ag^+^ binding,^9,14^ SP2 CSPs can be influenced by the change of structure in the backbone of the peptide and by the Ag^+^ binding. Indeed, amide protons, within an α-helical arrangement, are involved in the hydrogen-bond formation that occurs between them and the carbonyl oxygen on *i + 4* position. At the surface of an α-helical structure, a particular residue at a given position *i* has its side chain on the same face of the helix as residue *i + 3* and *i + 4*. This consideration helps to evaluate the possible Ag^+^ binding site of SP2 peptide. The high CSP values for 90Met and 91Met appearing in the first group and confirmed by the second one, suggest that these two residues are involved in Ag^+^ binding. For 90Met, this is also confirmed by previous studies.^10,14^

**Fig. 3:**
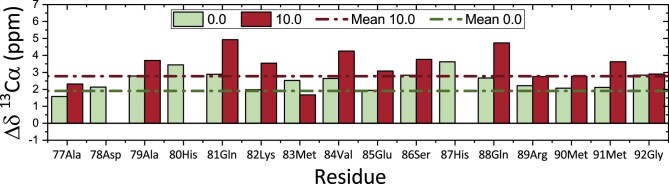
Difference between random coil ^13^C_α_ chemical shift^17^ and ^13^C_α_ chemical shift of 0.0:1.0 and 10.0:1.0 Ag^+^: SP2 ^1^H-^13^C HSQC spectra, respectively, in green and red.

Previous studies show that ^1^H_α_ and ^13^C_α_ are between the most helpful nuclei in the determination of the position of the binding site.^15^ However, in general, carbons are buried in the peptide structure, especially carbons in α position. Because ^13^C signals are dependent on dihedral angle,^11^ it is reasonable to hypothesize that the CSPs of C_α_ are mainly influenced by structural rearrangement of the SP2 upon silver binding. To support this hypothesis, the ^13^C chemical shift difference was calculated using tabulated random coil ^13^C values as a reference.^16^ This difference was calculated for both the 0.0:1.0 and 10.0:1.0 Ag^+^: SP2 ^1^H-^13^C HSQC titration spectra and is reported in Fig. 3.

Even in its apo-form, the SP2 peptide seems to be structurally organized and not completely random coil. All the chemical shift differences in Fig. 3 are positive, but in the 10.0:1.0 point they are greater than in 0.0:1.0. This can be interpreted as a strengthening and stabilization of a preexisting secondary structure upon silver binding.

**Fig. 4:**
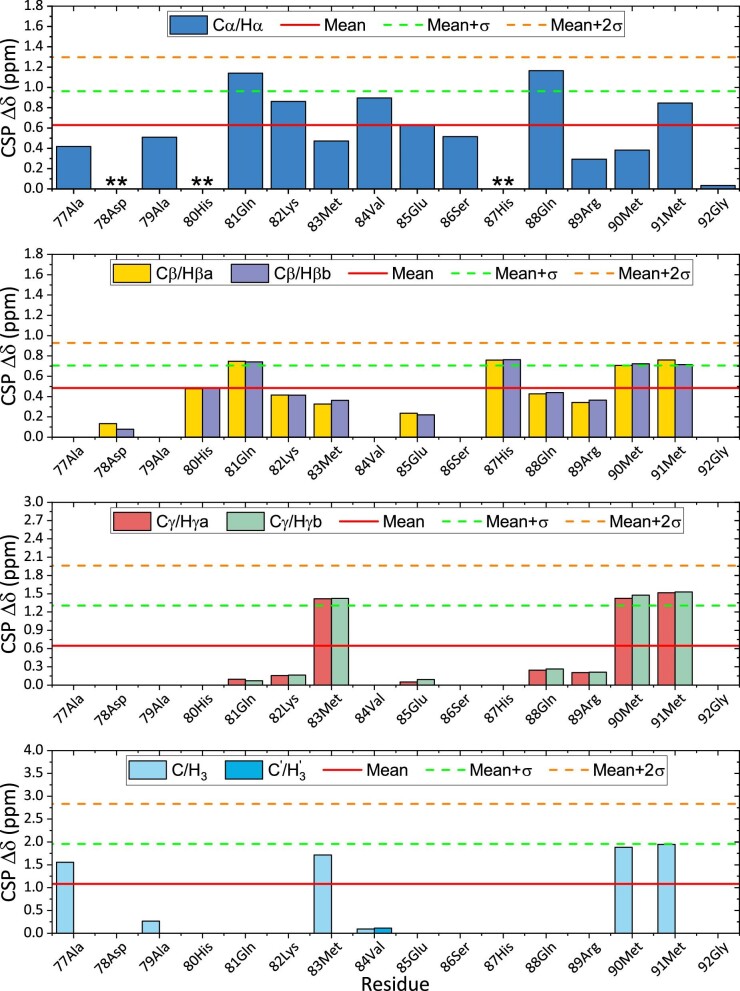
^1^H-^13^C HSQC CSPs histogram graphs put in the following order from top to bottom: first (C_α_/H_α_), second (C_β_/H_β_), third (C_γ_/H_γ_), and fourth (CH_3_) group of signals (Supplementary Fig. S3). (**∗∗**) Missing cross-peak not visible in the spectra during the titration.

The group subdivision, based on the atoms connected to the carbon of interest, allows comparison of similar carbons on different residues discarding the influence of other variables on the analysis. In Fig. 4, all the histograms reporting the CSPs values for each of the ^1^H-^13^C HSQC signals are grouped. Those residues that have no bars or labels indication in their position are not part of the group of signals considered. Mean and standard deviation (σ) are also plotted on each graph to allow the discrimination between weak, medium, and strong movers.

**Fig. 5:**
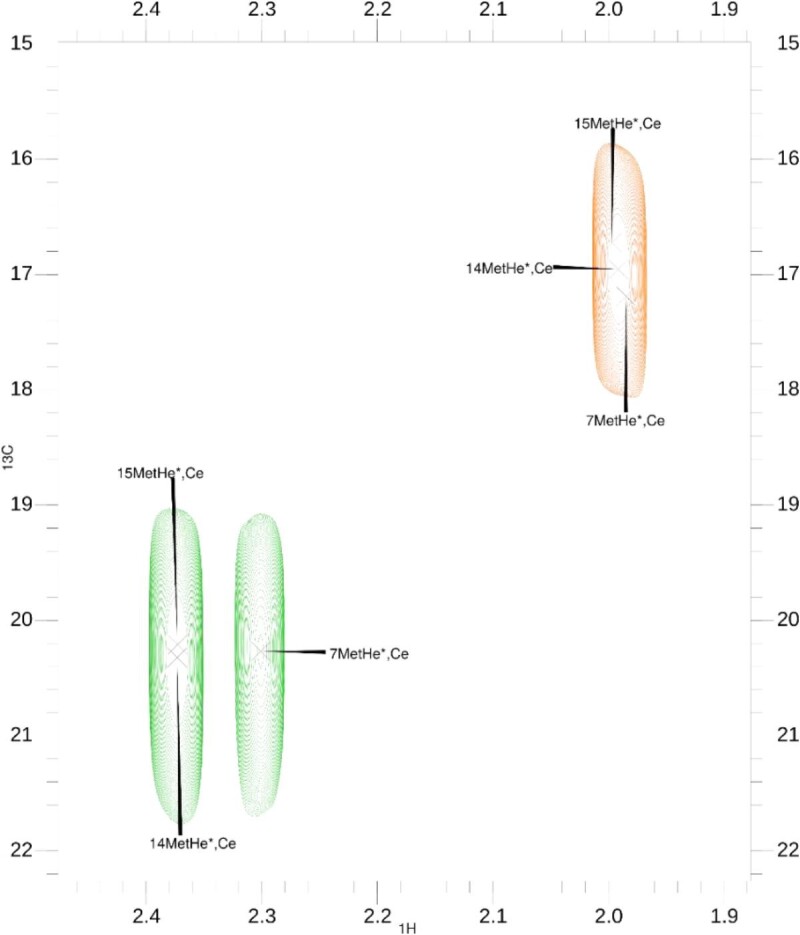
^1^H-^13^C HSQC spectra overlap. Close up on ^1^H-^13^C HSQC ^13^C_ε_/^1^H_ε_ region of methionine residues. Cross-peaks of the 0.0:1.0 and 10.0:1.0 Ag^+^: SP2 spectrum are in orange and green, respectively. Figure made with CcpNMR Analysis V2.

CSPs, reported in Fig. 4, show that the main residues affected by Ag^+^ binding are 83Met, 90Met, and 91Met. Moreover, the three C_ε_/H_ε_ cross-peaks of these residues overlap in the 0.0:1.0 spectrum but they shift during the titration with Ag^+^, leading to a separation in two groups in the 10.0:1.0 spectrum (Fig. 5). This is an additional suggestion that, possibly, two different regions are involved in the silver binding by the SP2 peptide, both using a methionine residue to coordinate the metal ion.

**Fig. 6:**
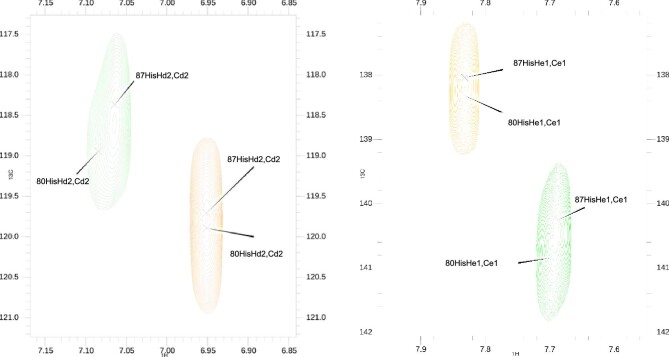
^1^H-^13^C HSQC spectra overlap. Close up on ^1^H-^13^C HSQC ^13^C_δ_/^1^H_δ_ region of histidine residues (left). Close up on ^1^H-^13^C HSQC ^13^C_ε_/^1^H_ε_ region of histidine residues (right). Cross-peaks of the 0.0:1.0 and 10.0:1.0 Ag+: SP2 spectra are in orange and green, respectively. Figures made with CcpNMR Analysis V2.

The involvement of histidine residues in silver binding by the SilE protein is suggested in literature.^9,10,14,17^ Looking at the second group of CSPs in Fig. 4, 87His shows a higher value compared to 80His, which can be barely located in the weak movers region. Although there is a large difference in the CSPs of carbons in β position of 80His and 87His (Fig. 6), these residues both show an interesting movement in C_δ_/H_δ_ and C_ε_/H_ε_  ^1^H-^13^C HSQC region of cross-peaks. These regions are magnified in the right part of Fig. 6.

On the left side of Fig. 6, C_δ_/H_δ_ cross-peak moves at higher ^1^H and lower ^13^C chemical shifts when Ag^+^ is added. However, on the right side of Fig. 5, C_ε_/H_ε_ cross-peak moves at lower ^1^H and higher ^13^C chemical shifts after addition of silver to the solution. Assuming that histidine residues in SP2 are involved in complex formation, the metal coordination should take place with the participation N_δ_ of the imidazole group of side chain of each histidine as suggested by the evidence in Fig. 6. This assumption was made because N_δ_ in the imidazole ring is connected to C_ε_, which is de-shielded (higher chemical shift) upon Ag^+^ addition, while C_δ_ shows an opposite behavior indicating that N_ε_ probably is not involved in silver binding.

**Fig. 7:**
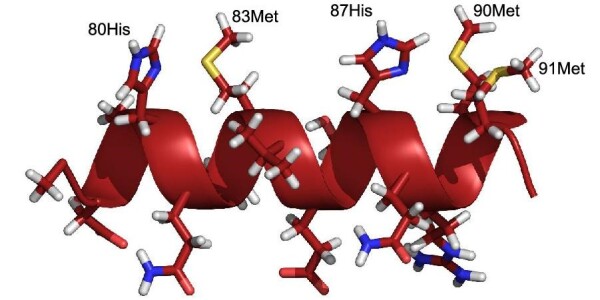
Cartoon model of the SP2 peptide with an α-helical secondary structure. The backbone and the carbon atoms are depicted in dark red, nitrogen and sulfur atoms are in blue and yellow, respectively. 80His, 83Met, 87His, 90Met and 91Met residues are highlighted to facilitate the recognition of their spatial position.

Figure 7 shows the putative secondary α-helix structure of SP2. By a visual inspection, it can be seen that the 80His, 83Met, 87His, 90Met, and 91Met residues are on the same face of the peptide. This can be expected also by looking at their position in the primary amino acid sequence. Indeed, they are at a *i + 3* or *i + 4* distance with respect to each other, considering *i* as the position of a reference amino acid.

**Fig. 8:**
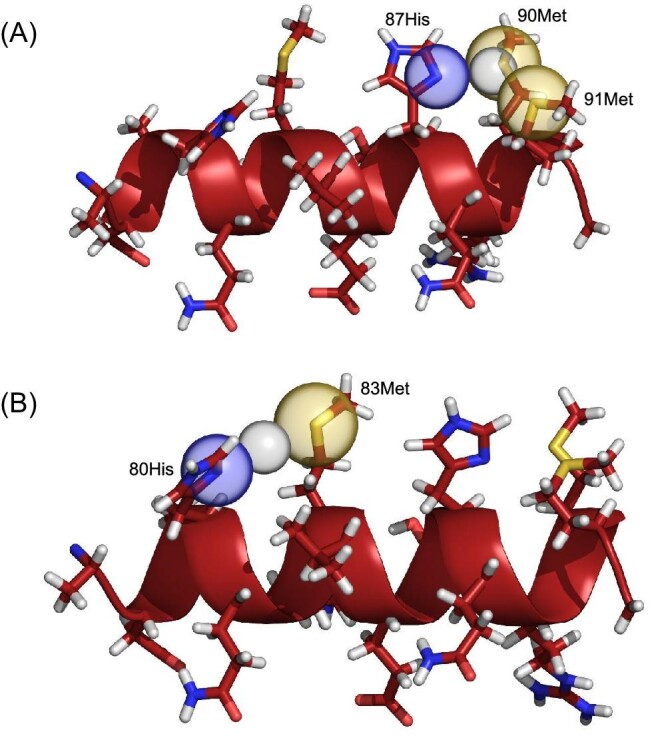
Cartoon model of the α-helical secondary structure of the SP2 peptide binding the Ag^+^ cation (gray). The backbone and the carbon atoms are depicted in dark red and nitrogen and sulfur atoms are in blue and yellow, respectively. (**A**) 87His, 90Met, and 91Met residues involved in the binding are highlighted to facilitate the recognition of their spatial position. They describe a triangle in which is located the Ag^+^, presumably forming a distorted trigonal planar coordination geometry. (**B**) 80His and 83Met residues involved in the binding are highlighted too. They face each other with the Ag^+^ in between, presumably forming a linear coordination geometry. Nitrogen and sulfur donors in the putative binding site are represented as transparent sphere with their VdW radius (N_VdW_ = 1.55 Å, S_VdW_ = 1,80 Å, Ag^+^_VdW_ = 1.15 Å).

Moreover, these sequences of amino acids contain the SilE silver binding motif His-X-X-Met suggested in literature^9,17^ where X is a proteogenic amino acid. The α-helix structure represented in Fig. 7 gives a clear idea of how the residues are arranged in space. In particular, there are two putative binding sites for silver on the same face of the peptide, both composed of histidine and methionine residues. Figure 8 shows two models of Ag^+^ bound to SP2, simply obtained from the CSPs data.

The first 3D model of SP2 in Fig. 8A was created to show the first putative binding mode. From the TOCSY CSPs data (Supplementary Figs. S1 and S2), 90Met and 91Met show the highest CSPs for N-H/H_α_ cross-peaks. From the ^1^H-^13^C HSQC CSPs data in Fig. 5, 87His C_β_/H_β_ cross-peaks are in the region of the medium movers together with the C_β_/H_β_ cross-peaks of 90Met and 91Met. In addition, C_γ_/H_γ_ and C_ε_/H_ε_ cross-peaks of methionine residues show the highest CSPs among all the other residues. C_γ_ and C_ε_ in the side chain of methionine are directly connected to the sulfur (S_δ_) that is presumably involved in the Ag^+^ binding. Thus, higher CSPs for them are indicative of local environmental change. Moreover, in the imidazole ring of 87His, C_ε_ gets de-shielded upon the addition of silver, while C_δ_ does not (Fig. 6). The Ag^+^ cation is at the center of the triangle described by 87His, 90Met, and 91Met. The side chain of these residues were oriented to minimize the distance between the N_δ_ of the imidazole ring of 87His and Ag^+^, as well as the distance between the S_δ_ of 90Met and 91Met and Ag^+^. The three ligand donors describe a triangle in which is located the Ag^+^, presumably forming a distorted trigonal planar coordination geometry. In previous studies, methionine residues are reported to coordinate Ag^+^ ions in nonlinear geometries with Ag^+^: M_2_ and Ag^+^: M_3_ stoichiometries in the multicopper oxidase CueO,^18^ whereas CusF coordinates silver ions through histidine, methionine and tryptophan residues.^19^

The second 3D model of SP2 in Fig. 8B was created to show the second putative binding mode. The second binding region that will be discussed is the one described by 80His and 83Met. From the TOCSY CSPs data (Supplementary Figs. S1 and S2), 80His N-H/H_α_ cross-peak is in the region of the weak movers, like the H_α_/H_β_ cross-peaks of 83Met. However, C_γ_/H_γ_ and C_ε_/H_ε_  ^1^H-^13^C HSQC cross-peaks of 83Met residue have CSPs in the region of medium and weak movers, respectively (Fig. 5). Thus, from the considerations made for the first model, we can assume that the sulfur in the side chain of 83Met is presumably involved in the Ag^+^ binding. Moreover, in the imidazole ring of 80His, C_ε_ gets de-shielded upon the addition of silver, while C_δ_ does not (Fig. 6). In this second model, complexation of the Ag^+^ ion can occur in a linear fashion (Fig. 8B).

**Fig. 9:**
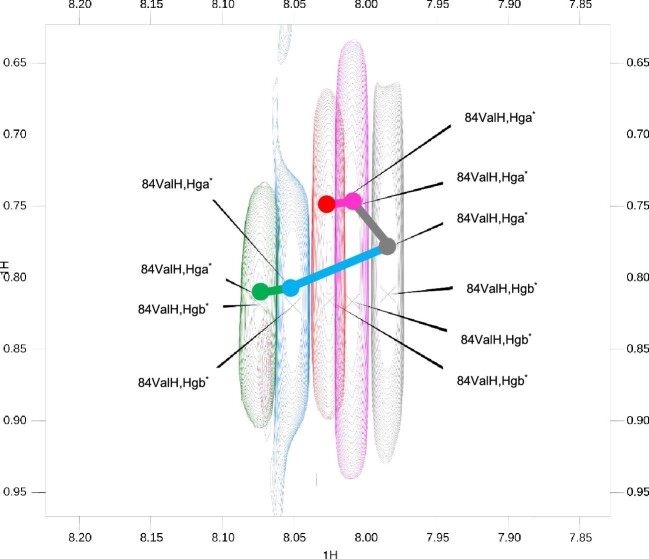
Titration TOCSY spectra overlapped. The zero point is the spectrum in green, the 0.1:1.0 in blue, the 0.5:1.0 in gray, the 3.0:1.0 in magenta and the 10.0:1.0 in red. The path of 84Val N-H/H_γa_ cross-peak is depicted using lines and points colored according to the spectra color scheme.

On the assumption that there are two binding regions for Ag^+^ to SP2, it is expected that this peptide can bind more Ag^+^ ions, each with different affinity. This would lead to a two steps binding process; in the first the silver ion binds the high affinity site called the primary site and in the second it binds the low affinity region called the secondary site. This process can be detected by looking at the titration spectra. If there is a single binding mode, the movement of the peaks should follow a straight line as the available site is being saturated. If there are multiple binding sites, the peak should follow a curved line with change of direction because the second binding mode would have a different effect on the chemical shift compared to the primary interaction.^11^ As an example, data in Fig. 9 highlight the nonstraight route followed by N-H/H_γ_ cross-peak. Figure 9 shows how the N-H/H_γ_ cross-peak of valine residue moves during the titration. It follows a straight line from the 0.0:1.0 point (green) to the 0.5:1.0 point (gray). After that point, it changes direction. This evidence indicates that the SP2 peptide undergoes two different binding modes upon the addition of silver.

**Fig. 10:**
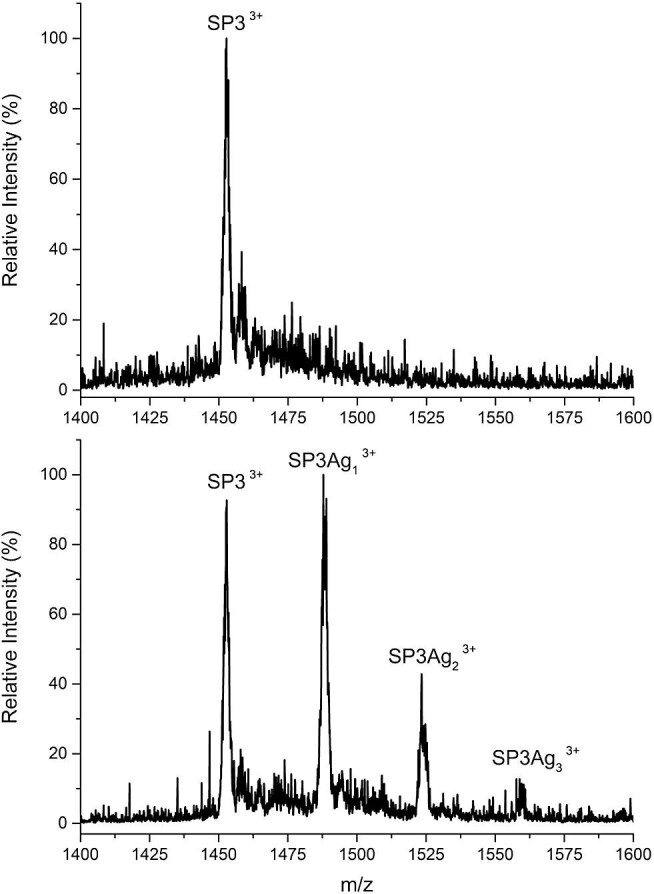
ESI–MS spectra acquired in positive ion mode for a 100 μM solution of SP3 (top) and in presence of Ag^+^ (bottom). Species attributed to different SP3Ag_n_ complexes with *n* = 1–3 are detected. All the solutions analysed were at pH = 7.0 in 9 mM ammonium acetate buffer.

Another issue remaining unsolved is the exact stoichiometry and binding affinity of longer SilE model peptides with silver. Indeed, previous studies only show that isolated short MX_2_H and HX_2_M motifs can bind Ag^+^ ions with strong affinity.^10,14^. For this reason, here we use a spectrometric approach aimed at determining stoichiometry and binding affinities of silver ions for the longer (19 amino acids) SilE model peptide SP3. Therefore, we examined the interaction between SP3 and silver ions through ESI–MS. The ESI–MS spectra were obtained from four solutions of the SP3 peptide. All the solutions were 100 μM in SP3 peptide, with increasing concentration of AgNO_3_. The first solution contained the *apo*-SP3, the other solutions were prepared at a Ag^+^: SP3 concentration ratio of 0.5:1, 1:1, and 2:1, respectively. It is important to highlight that the *apo*-SP3 peptide is mainly detected as a dimer by ESI–MS (Fig. 10), most likely due to electrostatic interactions occurring mainly in the gas phase rather than to truly existing species in solution, as it is often the case for peptides.^20^ Furthermore, in a previous paper,^14^ it is reported that an A2 model peptide, which shares part of the sequence with our SP3 model peptide, is in a monomeric form in solution. In any case, as the purpose of our investigation is to determine the stoichiometry of the Ag^+^: SP3 complex by varying the metal: peptide concentration ratio, the MS detection of SP3 as a dimer does not hinder the following investigations. Indeed, upon Ag^+^ addition, a change in the peak pattern in the ESI–MS spectra can be seen (Fig. 10). Assumed that the ESI–MS spectrum of SP3 changes as Ag^+^ is added (until Ag^+^: SP3 = 2:1) to the solution, a family of (SP3)Ag_n_ species with *n* up to 3 is detected when Ag^+^ is present. Because the formation of pseudo-molecular ions (adducts) with metal ion is common in ESI–MS,^21^ and they form rarely from true equilibria in solution, it is necessary to distinguish which one of the forms present in the ESI–MS spectra is really present in the sample and not only forming in the gas phase during the ionization process.^22^ To determine which of the detected SP3Ag_n_ complexes faithfully exist in solution, SP3 was incubated with increasing concentrations of Ag^+^ at pH 7.0. The concentrations of free SP3 and Ag^+^–SP3 complexes, calculated from the total SP3 and Ag^+^ added as well as from the relative peak areas, were plotted as a function of the amount of silver added to the solution (Supplementary Fig. S4). All the detected positively charge states were considered for calculating the relative amounts of free SP3 and Ag^+^–SP3 complexes. It was assumed that the total signal response for each individual species was proportional to the concentration of that species in the gas phase, and by extension, in solution. The estimation of the dissociation constant (calculated as reported in the supplementary information, Supplementary Fig. S2) indicates that the adducts containing one or two silver ions are the most stable. In particular, the magnitude of the *K*_D1_ is lower than 1.0 μM, as previously reported on a similar but shorter SilE model peptide (≅0.2 μM).^14^ On the contrary, *K*_D2_ is clearly higher than *K*_D1_ but still in the micromolar range, with a value that differs from previous studies on similar systems (≅3.2 μM).^14^ This confirms that the peptide analysed have two silver binding sites with different binding affinity.

## Conclusion

The CD spectra of *apo*-SP2 and *apo*-SP3 demonstrate that they exist as random coil peptides. After the addition of silver ions, they gain a secondary α-helix structure and an excess of Ag^+^ ions (10:1 Ag^+^: SP2 ratio and 8:1 Ag^+^: SP3 ratio, respectively) causes a complete folding and stabilization of the two peptides.

Looking at the ^13^C chemical shift difference of *apo*-SP2 it seems that it exists as a secondary structured peptide even before the addition of silver. It is possible that Ag^+^ ions have an effect on the strengthening and stabilization of a preexisting secondary structure of the SP2 peptide. NMR titration with silver and CSP studies on SP2 here presented clearly show that the main residues influenced by the addition of Ag^+^ in solution are 80His, 83Met, 87His, 90Met, and 91Met. From the Met—C_ε_/H_ε_ cross-peak region in ^1^H-^13^C HSQC spectra, a clear movement of methionine cross-peaks can be seen during the titration with Ag^+^. Also, C_δ_/H_δ_ and C_ε_/H_ε_  ^1^H-^13^C HSQC cross-peaks of histidine residues show a perturbation upon the addition silver. Thus, the SP2 peptide is involved in silver binding and it exerts this capability through its histidine and methionine residues. These findings led to the assumption that SP2 has two binding sites, the first made up of a histidine (80His) and a methionine (83Met) residue involved in the silver coordination with their side chains, forming a linear complex 80His-N_δ_–Ag^+^–S_δ_-83Met. The second binding site is made up of a histidine (87His) and two methionine (90Met and 91Met) residues involved, with their side chains, in a distorted trigonal planar complexation of Ag^+^. Furthermore, cross-peaks in the NMR titration spectra changed their path direction after the 0.5/1.0 (Ag^+^/SP2) concentration was reached. This suggests that SP2 undergo multiple binding modes.

Our study was then focused on SP3, a longer SilE model peptide containing the MX_2_H and HX_2_M motifs, due to the lack of knowledge regarding the exact stoichiometry and binding affinity for the interaction between longer SilE model peptides and silver ions. Indeed, ESI–MS analysis on the SP3 model peptide revealed that the latter interacts with Ag^+^ ions. We were able to distinguish the SP3Ag_n_ complexes actually present in the sample from the ones only forming in the gas phase during the ionization process. The dissociation constants of SP3 were estimated from the ESI–MS spectra by plotting the concentration of SP3 and SP3Ag_n_ complexes as a function of the concentration of Ag^+^ added in solution (Supplementary Fig. S4). The dissociation constant *K*_D1_ is found to be lower than 1.0 μM whereas the *K*_D2_ is found to be 24.5 μM.

As reported by Asiani *et al*.,^9^ SP2 and SP3 peptides contain the silver binding motifs that are located at the center of the primary full-length SilE sequence in a core providing initial nucleation sites for Ag^+^-induced folding. Hence, the data here reported represent a structural and functional basis to unveil the mechanism of interaction between silver ions and the SilE protein, thereby providing important insights for interpreting one out of the possible mechanisms of silver resistance acquired by *S. typhimurium*. These findings anticipate the setup of experimental models of greater complexity in order to figure out pharmacological strategies designed to overcome such a microbial resistance that poses a challenge in several clinical areas such as ophtalmology, surgery and dentistry.

## Supplementary Material

mfad015_Supplemental_File

## Data Availability

The data underlying this article will be shared on reasonable request to the corresponding author.
