# Supplementary material for: Insights into the binding of Ag ions with SilE model peptides: an NMR and MS coupled approach
Source: Metallomics. 2023 Mar 13;15(4):mfad015. doi: 10.1093/mtomcs/mfad015 (PMC10080550; doi:10.1093/mtomcs/mfad015)
Supplement: mfad015_Supplemental_File [file mfad015_supplemental_file.docx]

**SUPPLEMENTARY INFORMATION**

**Insights into the binding of Ag ions with SilE model peptides: an NMR and MS coupled approach**

**Gabriele Antonio Zingale^1$^, Valentina Oliveri^2^ and Giuseppe Grasso^2^**

^1^ IRCCS-Fondazione Bietti, Rome, Italy

^2^ Department of Chemical Sciences, University of Catania, Catania, ITALY

^$^Author to whom all the correspondence should be addressed:

*gabriele.zingale@phd.unict.it*

*^1^H-^1^H TOCSY Chemical Shift Perturbations (CSPs): group classification.* The first group of CSPs was made of all the TOCSY cross-peaks associated with the interaction between the N-H and the Hα of all amino acids in the peptide chain. All of these protons have a similar environment in the backbone of the peptide, thus their CSP values can be grouped and compared. **Fig. S1** shows the CSP value for each N-H/H_α_ cross-peak. Mean and standard deviation (σ) are plotted and a further discrimination is then based on the CSP value. Peaks that show a CSP value in the region between the mean and mean plus one standard deviation are defined as weak movers. Those that are in the region between mean plus σ and 2σ are defined as medium movers. Finally, those that are in region between mean plus 2σ and 3σ are defined as strong movers. This is valid for all the considerations that are made in the text. The second group of signals was made of all the TOCSY cross-peaks associated to CH_2_ surrounded by a CH and a CH_2_. This is typical for the beta position of the glutamine (Gln), glutamic acid (Glu), lysine (Lys), arginine (Arg) and methionine (Met) residues. With similar criteria applied, the third group of signals was made of all the TOCSY cross-peaks associated to CH_2_ surrounded by two CH_2_. This is typical for the gamma position of Arg and Lys, and for delta position of Lys residues. All of these protons have a similar environment when considered alone, thus their CSP values can be grouped and compared since they have a common “starting point”. The fourth group of signals was made of all the TOCSY cross-peaks associated to CH_3_. **Fig. S2** shows the CSPs values for each group of signals. Those residues that have no bars or labels indication in their position are not part of the group of signals considered. Mean and standard deviation (σ) are also plotted on each graph to help in the discrimination between weak, medium and strong movers.


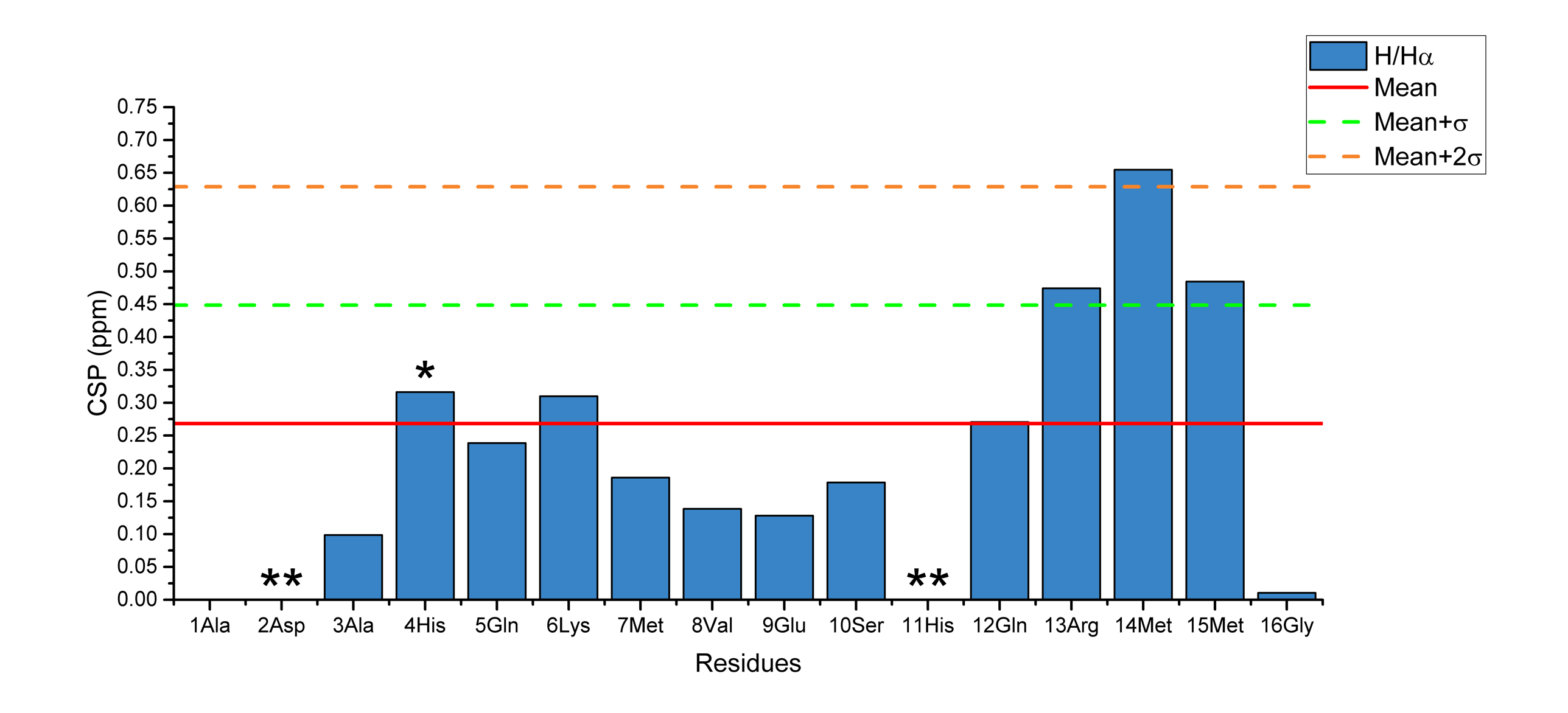


**Fig S1.** Histogram graph showing CSP values for each NH/H_α_ cross-peak (top). Example of a structure of an amino acid residue in the chain used to highlight the ^1^H involved in the analysis (bottom). (**∗**) 4His H/H_α_ has the last point at 0.5:1.0 instead of 10.0:1.0. (**∗∗**) Missing cross-peak not visible in the spectra during the titration.


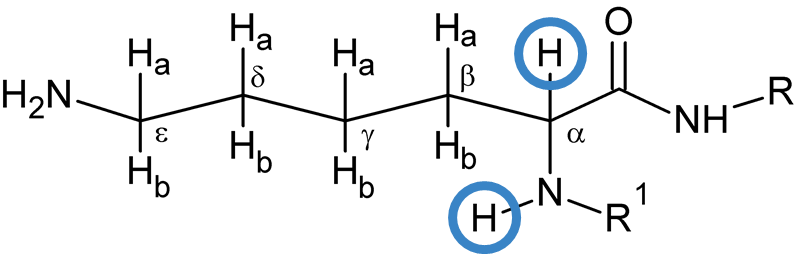


**Fig S2.** TOCSY CSPs histogram graphs put in the following order from top to bottom: first (N-H/H_α_), second (CH_2_ surrounded by a CH and a CH_2_), third (CH_2_ surrounded by two CH_2_) and fourth (CH_3_) group of signals. (**∗**) In the first graph, 4His H/H_α_ has the last point at 0.5:1.0 instead of 10.0:1.0. (**∗**) In the second graph, 15Met CSPs have the starting point at 0.1:1.0 instead of 0.0:1.0. (**∗∗**) In the first graph identifies missing cross-peak not visible in the spectra during the titration.





*^1^H-^13^C HSQC Chemical Shift Perturbations (CSPs)*. The procedure followed to form four different carbon groups is similar to the one previously seen for TOCSY CSPs groups, but with slightly different criteria. The first group of CSPs was made of all the HSQC cross-peaks associated to the interaction between the C_α_ and the H_α_ of all amino acids in the peptide chain. All of these carbons have a similar environment in the backbone of the peptide, thus their CSP values can be grouped and compared. Indeed, they are connected to one amidic nitrogen, two carbons and one hydrogen. The second group of signals was made of all the HSQC cross-peaks associated to CH_2_ connected to other two carbons. This is typical for the C_β_ of the aspartic acid (Asp), histidine (His), glutamine (Gln), glutamic acid (Glu), lysine (Lys), arginine (Arg) and methionine (Met) residues. The third group of signals was made up of all the HSQC cross-peaks associated to CH_2_ connected to two carbon or one carbon and one sulphur. This is the case of the gamma position of Gln, Lys, Met, Glu and Arg residues. The fourth group of signals was made of all the HSQC cross-peaks associated to CH_3_ connected to a carbon or a sulphur. This is the case of C_β_ of alanine (Ala), C_γ_ of valine (Val) and C_ε_ of Met. The exception of sulphur is made because it has almost the same electronegativity of carbon (sulphur 2.58 and carbon 2.55). Similar to the group subdivision of TOCSY cross-peaks, HSQC groups of cross-peaks relies upon the environmental equivalence between each group components. **Fig. S3** shows a graphical representation of the group classification.


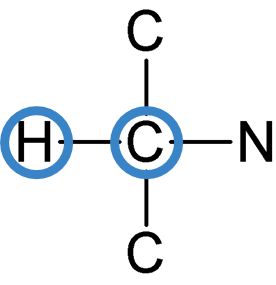

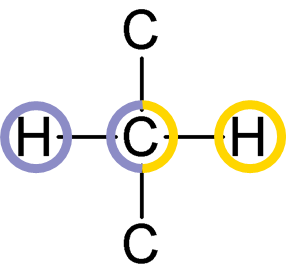

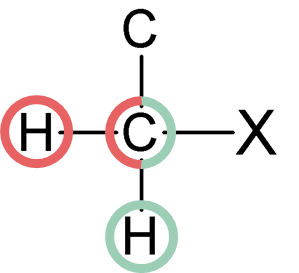

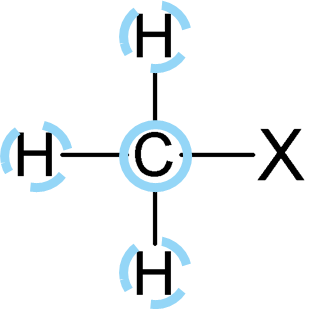


**Fig S3.** General exemplification of different group of carbons. From left to right, carbon of the first (C_α_/H_α_), second (C_β_/H_β_), third (C_γ_/H_γ_) and fourth (CH_3_) group of signals. X indicates any atom between carbon and sulphur. The colour scheme is the same used in Fig.4 in the main text.

*ESI-MS spectrometry.* The concentrations of free SP3 and Ag^+^–SP3 complexes, calculated from the total SP3 and Ag^+^ added as well as from the relative peak areas (normalized to the total ion current), were plotted as a function of the amount of silver added to the solution (Fig. S4). All the detected positively charge states (3+ and 4+) were considered for calculating the relative amounts of free SP3 and Ag^+^–SP3 complexes. It was assumed that the total signal response for each individual species was proportional to the concentration of that species in the gas phase, and by extension, in solution. From Fig. S4, the total Ag^+^ concentration at which the amount of free SP3 was equal to the amount of singly complexed S3 was determined to be equal to 100 µM. At this point, the concentration of free SP3 was equal to the concentration of singly complexed SP3. Assuming an equivalence of concentrations and activities, K_D1_ is equal to the concentration of free Ag^+^ in solution:

$$K_{D1}=\frac{\left[ SP3 \right][{Ag}^{+}]}{[SP3\cdot{Ag}^{+}]}=\left[ {Ag}^{+} \right] (1)$$

Similarly, further dissociation constants K_Dn_ can be obtained from the equation (1) as the concentration of free Ag^+^ in solution when the amount of SP3Ag_n-1_ is equal to the amount of SP3Ag_n_:


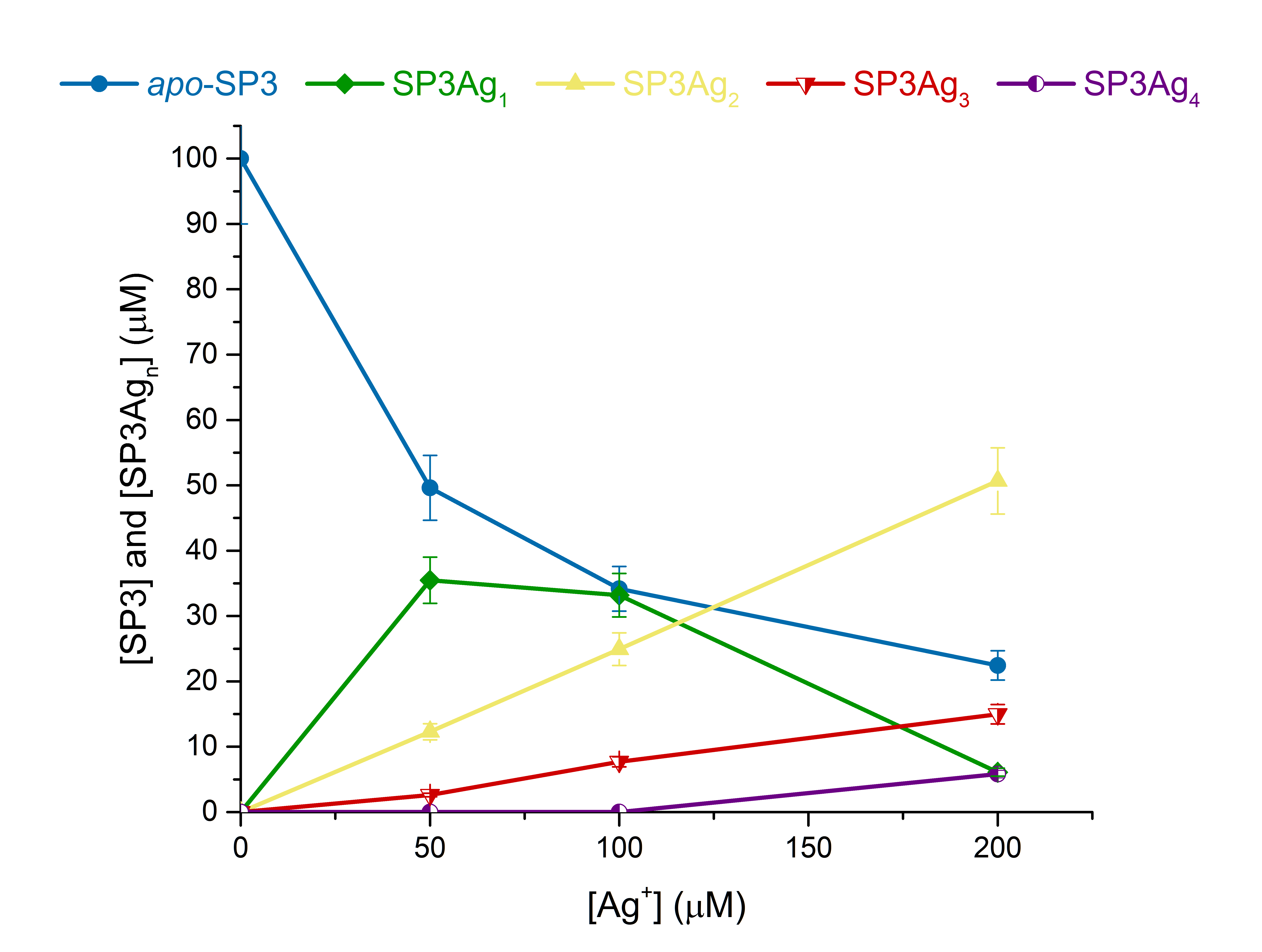


**Fig S4.** Monitoring the SP3Ag complexes obtained by ESI-MS measurements. Only the SP3Ag_n_ species with n = 1 and n = 2 were due to specific SP3-Ag interactions (lines with different colours). Error bars were set to the 10% of data.

$$K_{Dn}=\frac{\left[ SP3\cdot(n-1){Ag}^{+} \right][{Ag}^{+}]}{[SP3\cdot n{Ag}^{+}]}=\left[ {Ag}^{+} \right] (2)$$

|  | **K_D1_** | **K_D2_** |
| --- | --- | --- |
| **SP3** | < 1.0 µM | 24.5 µM |

***Table 1.*** *Dissociation constants derived from ESI-MS data for the binding of Ag^+^ to SP3 peptide in 9mM ammonium acetate at room temperature and at pH = 7.0.*

Equations 1 and 2 were used to estimate K_D1_ and K_D2_ (Table 1) from the data in Fig. S4. The amount of free Ag^+^ in solution was calculated by subtraction of the total amount of Ag^+^ complexed by SP3 from the amount of Ag^+^ added. The estimation of the dissociation constant indicates that the adducts containing one or two silver ions are the most stable. In particular, the magnitude of the K_D1_ (Table 1) is lower than 1.0 µM. On the contrary, K_D2_ is clearly higher than K_D1_ but still in the micromolar range. This suggests that the peptide has two silver binding sites with different binding affinity. Clearly, all the other metal-peptide adducts containing more than two silver ions are formed only in the gas phase but do not really exist and/or their formation is negligible in solution.

| **Peak (m/z)** | **Charged state** | **Sequence** | **Mass (Da av.)** | **Assignment** |
| --- | --- | --- | --- | --- |
| 1089.60 | 4+ | AMNEHERAAVAHEFMNNGQ | 2156.322 | SilE_3 dimer plus two Na^+^ |
| 1116.60 | 4+ | AMNEHERAAVAHEFMNNGQ | 2156.322 | SilE_3 dimer plus two Na^+^ and one Ag^+^ |
| 1142.60 | 4+ | AMNEHERAAVAHEFMNNGQ | 2156.322 | SilE_3 dimer plus two Na^+^ and two Ag^+^ |
| 1452.73 | 3+ | AMNEHERAAVAHEFMNNGQ | 2156.322 | SilE_3 dimer plus two Na^+^ |
| 1488.73 | 3+ | AMNEHERAAVAHEFMNNGQ | 2156.322 | SilE_3 dimer plus two Na^+^ and one Ag^+^ |
| 1524.20 | 3+ | AMNEHERAAVAHEFMNNGQ | 2156.322 | SilE_3 dimer plus two Na^+^ and two Ag^+^ |
| 1560.27 | 3+ | AMNEHERAAVAHEFMNNGQ | 2156.322 | SilE_3 dimer plus two Na^+^ and three Ag^+^ |

***Table 2.*** *ESI-MS spectra assignment. The two main peaks at 1452.73 and 1089.60 m/z ratios belong to the same precursor that is a dimer of SP3 (3+ and 4+ charged state, respectively) with two Na^+^ ions.*


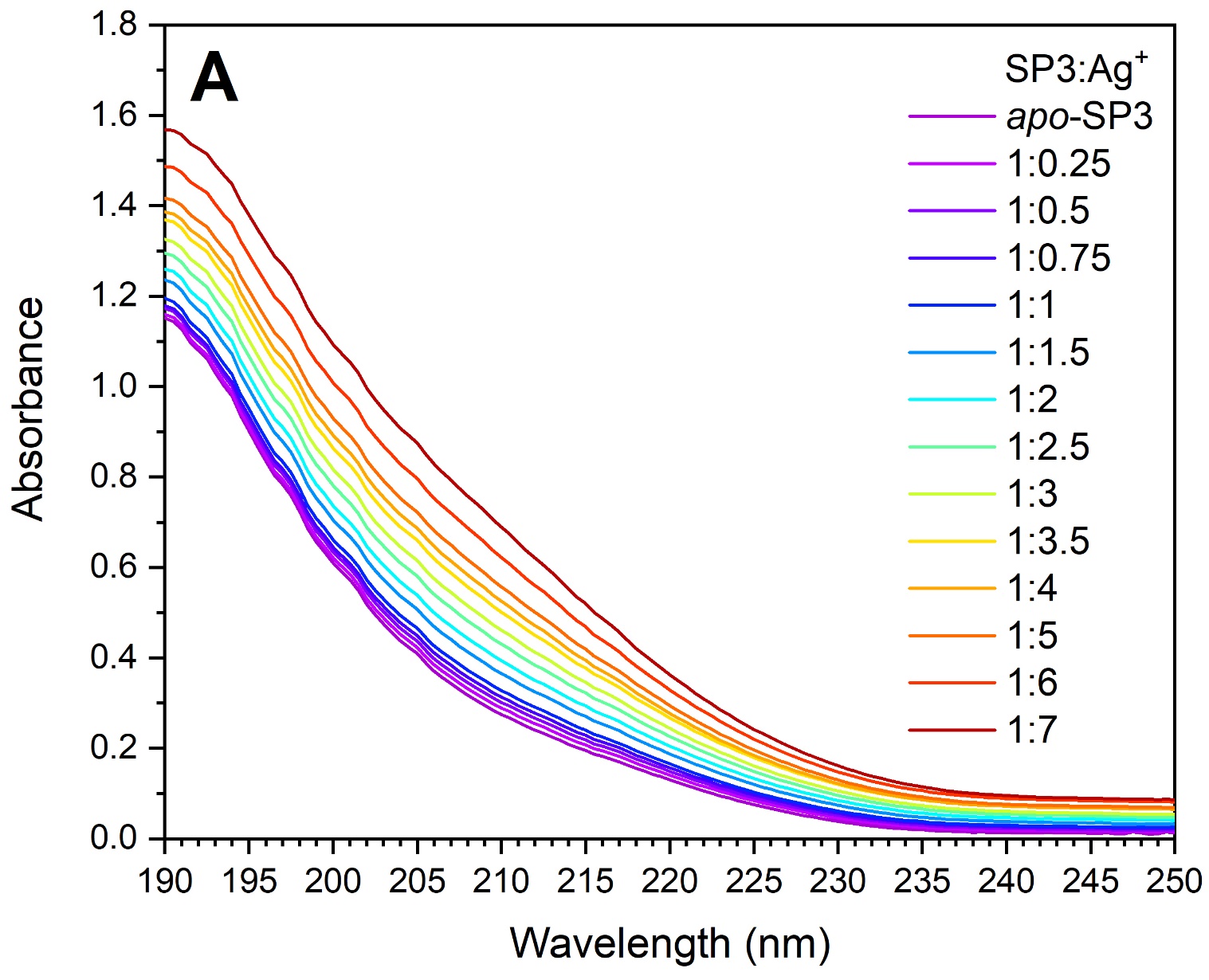


**Fig S5.** Absorption spectra of the SP3 solutions used for the CD experiments (**A**). Absorbance at 190 nm as a function of the equivalents of Ag^+^ added to the SP3 solution (**B**). Absorbance gap expressed as ΔA as a function of eq of Ag^+^ (**C**).


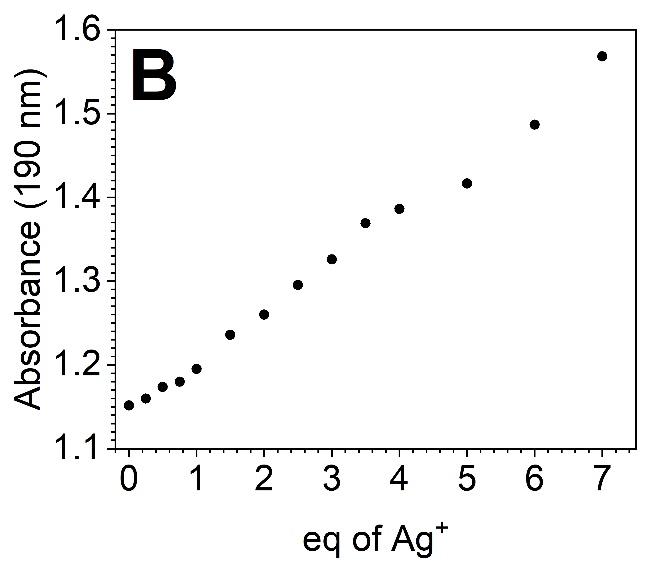

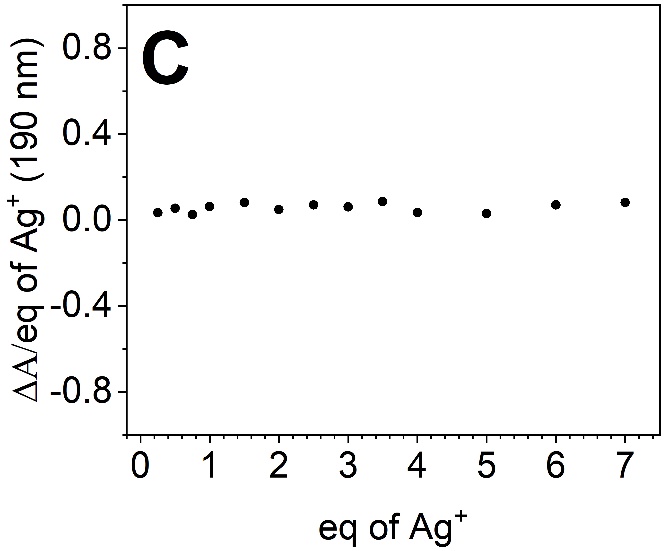

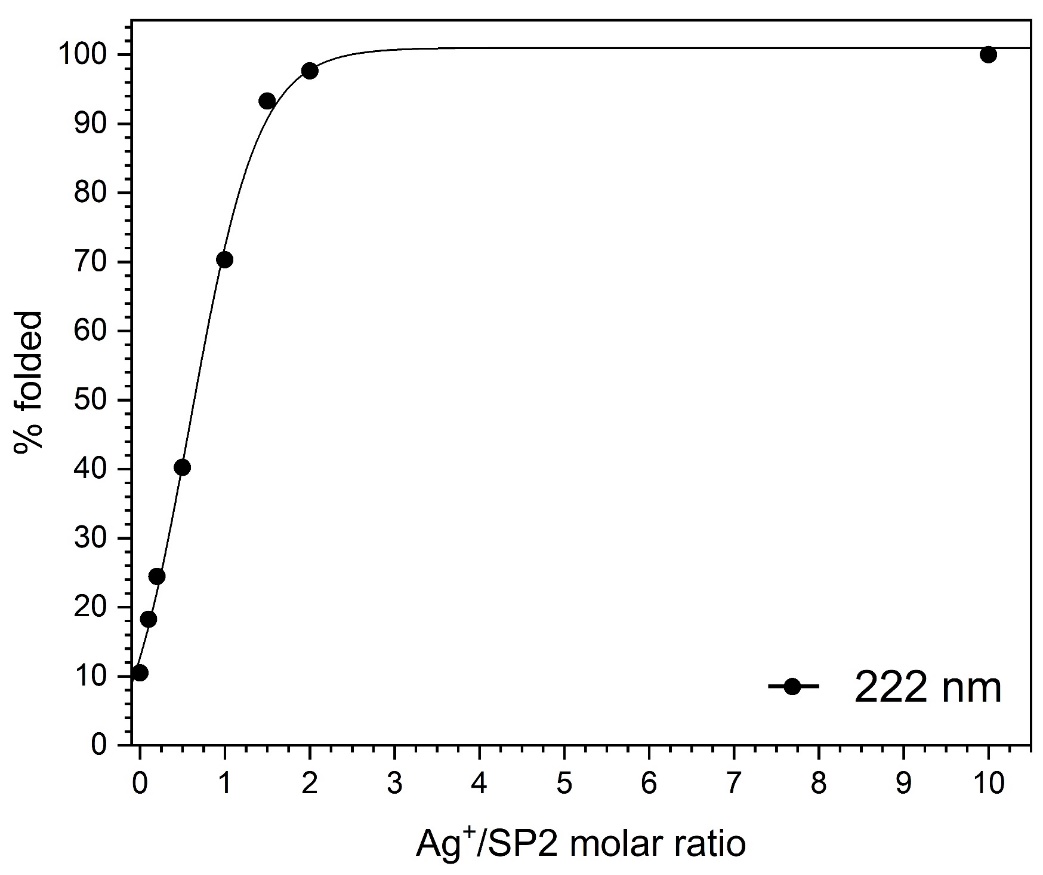


**Fig S6.** Plots of the CD signals as a function of the Ag^+^:SP2 and Ag^+^:SP3 molar ratios at 222 nm.


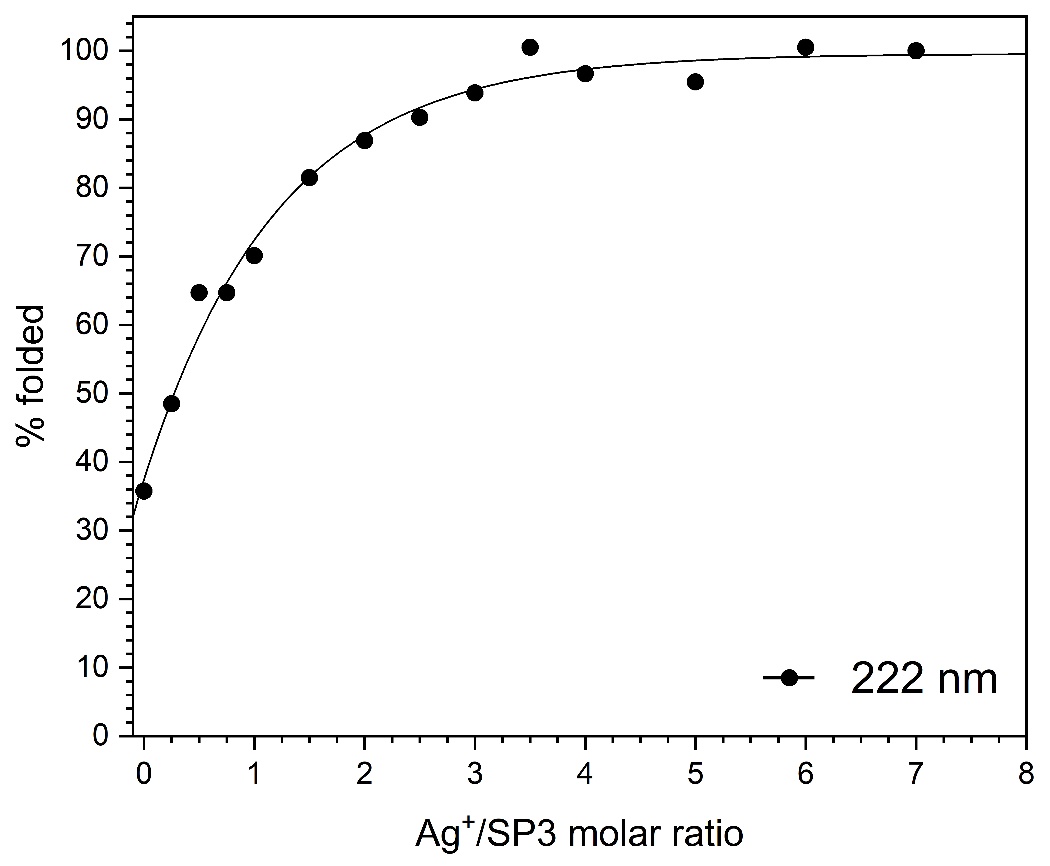

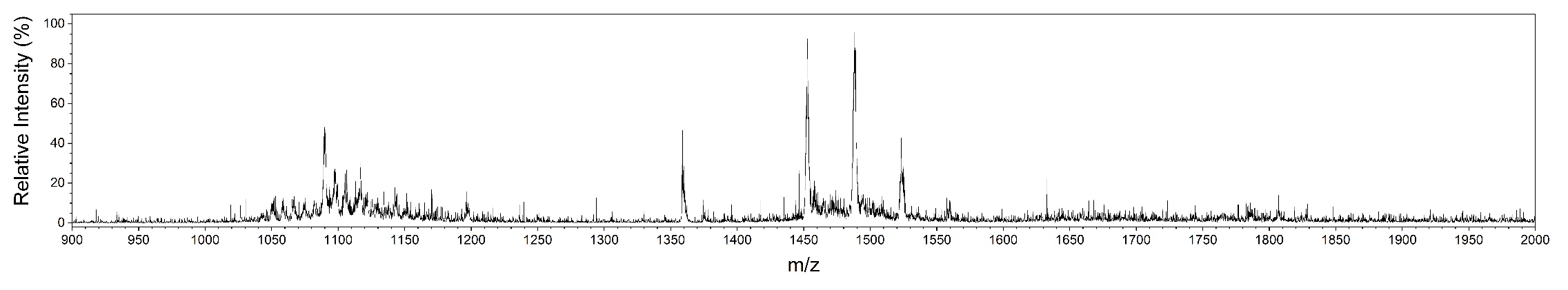

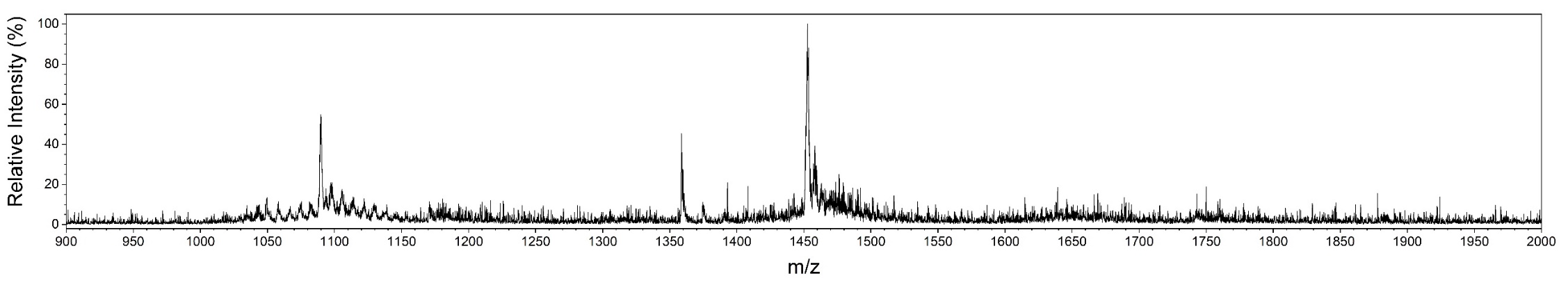


**Fig S7.** Full ESI-MS spectra acquired in positive ion mode for a 100 µM solution of SP3 (top) and in presence of Ag^+^ (bottom).
